# Supplementary material for: Saccharomyces boulardii (CNCM I-745) improves intestinal damage in sepsis by remodeling intestinal flora structure
Source: Front Cell Infect Microbiol. 2025 Jul 31;15:1602792. doi: 10.3389/fcimb.2025.1602792 (PMC12350329; doi:10.3389/fcimb.2025.1602792)
Supplement: Supplementary file 1 [file DataSheet1.zip › summary/summary/7_Differential_analysis/1_diff_abundance/6_Genus/Group.BW4_vs_AW4/Group.BW4_vs_AW4_diff_barplot.pdf]

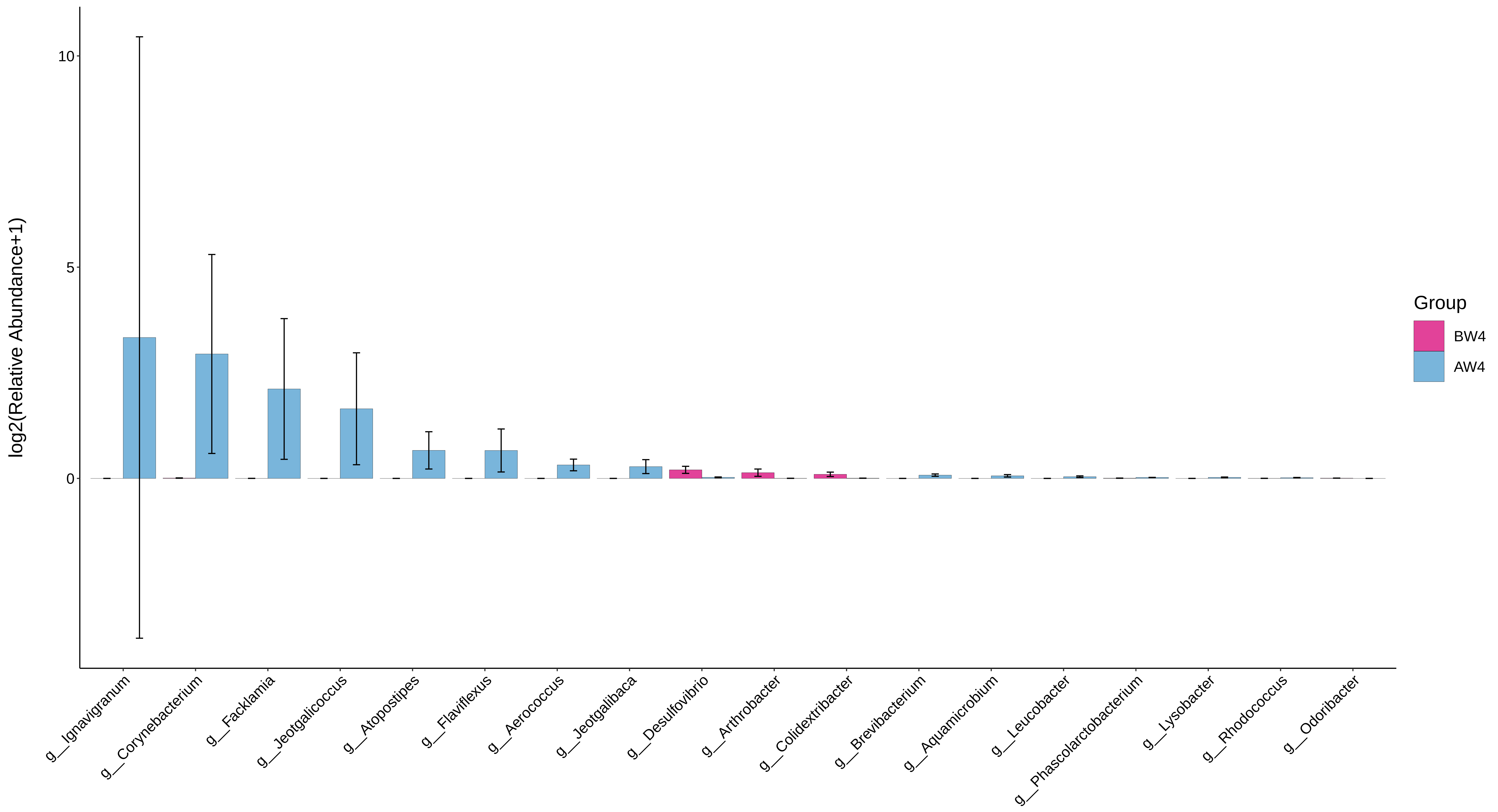

log2(Relative Abundance+1)
10
5
0
g__Ignavigranum
g__Corynebacterium
g__Facklamia
g__Jeotgalicoccus
g__Atopostipes
g__Flaviflexus
g__Aerococcus
g__Jeotgalibaca
g__Desulfovibrio
g__Arthrobacter
g__Colidextribacter
g__Brevibacterium
g__Aquamicrobium
g__Leucobacter
g__Phascolarctobacterium
g__Lysobacter
g__Rhodococcus
g__Odoribacter
Group
BW4
AW4
